# Supplementary material for: The perceived impact of multiple sclerosis and self-management: The mediating role of coping strategies
Source: PLoS One. 2021 Mar 11;16(3):e0248135. doi: 10.1371/journal.pone.0248135 (PMC7951896; doi:10.1371/journal.pone.0248135)
Supplement: Attachment — Submitted filename: PONE-D-20-29426_reviewer.pdf [file pone.0248135.s002.pdf]

PLOS ONE

Impact of multiple sclerosis and self-management: mediating role played by coping strategies

--Manuscript Draft--

**Manuscript Number:**	PONE-D-20-29426
**Article Type:**	Research Article
**Full Title:**	Impact of multiple sclerosis and self-management: mediating role played by coping strategies
**Short Title:**	Self-management in multiple sclerosis and coping
**Corresponding Author:**	Maciej Wilski, Ph.D.<br>Poznan University of Physical Education<br>Poznan, POLAND
**Keywords:**	multiple sclerosis; self-management; coping; disease impact
**Abstract:**	Low level of self-management in MS patients is considered to be a predominant factor that leads to poor rehabilitation efficacy. Studies focusing on the relationship between self-management and psychological variables that can be modified could contribute to expanding the knowledge needed to propose interventional programs aiming at patient activation. This study aimed to analyze whether coping strategies play a mediating role in the association between the perceived impact of multiple sclerosis (MS) and level of self-management in MS patients. The cross-sectional study included 382 MS patients. The patients completed the Multiple Sclerosis Self-Management Scale—Revised, Multiple Sclerosis Impact Scale-29, and Coping Inventory for Stressful Situations. The study hypothesis was evaluated using mediation analysis. The STROBE checklist specifically prepared for cross-sectional research was applied in this study for reporting. Results indicate that the emotion- and problem-focused strategies of coping can be treated as mediating the association between the MS impact and level of self-management in patients with MS. A negative relationship was found between the perceived MS impact and problem-oriented coping, while a positive relationship was found between problem-oriented coping and self-management. Furthermore, a positive relationship was found between the MS impact and emotion-oriented coping, while a negative relationship was found between emotion-oriented coping and self-management. The indirect role of the avoidance-oriented coping was not significant. Our study confirms the role played by coping strategies in patients' self-management. In MS, self-management determined by perceived MS impact can be controlled by decreasing emotional-coping while increasing problem-coping strategies. Our study imparts new knowledge regarding the potential interventions for improving the level of self-management in MS patients. It indicates that recognition of patients' illness perceptions as well as maladaptive coping strategies can help health professionals identify those who might be having lower level of self-management.
**Order of Authors:**	Maciej Wilski, Ph.D.
	Waldemar Brola
	Maciej Tomczak
**Additional Information:**	
**Question**	**Response**
**Financial Disclosure**<br><br>Enter a financial disclosure statement that describes the sources of funding for the work included in this submission. Review the submission guidelines for detailed requirements. View published research articles from *PLOS ONE* for specific	The author(s) received no specific funding for this work.

Powered by Editorial Manager® and ProduXion Manager® from Aries Systems Corporation

examples.<br><br>This statement is required for submission and **will appear in the published article** if the submission is accepted. Please make sure it is accurate.<br><br>**Unfunded studies**<br>Enter: *The author(s) received no specific funding for this work.*<br><br>**Funded studies**<br>Enter a statement with the following details:<br>• Initials of the authors who received each award<br>• Grant numbers awarded to each author<br>• The full name of each funder<br>• URL of each funder website<br>• Did the sponsors or funders play any role in the study design, data collection and analysis, decision to publish, or preparation of the manuscript?<br>• **NO** - Include this sentence at the end of your statement: *The funders had no role in study design, data collection and analysis, decision to publish, or preparation of the manuscript.*<br>• **YES** - Specify the role(s) played.<br><br>* typeset	
**Competing Interests**<br><br>Use the instructions below to enter a competing interest statement for this submission. On behalf of all authors, disclose any competing interests that could be perceived to bias this work—acknowledging all financial support and any other relevant financial or non-financial competing interests.<br><br>This statement **will appear in the published article** if the submission is accepted. Please make sure it is accurate. View published research articles from *PLOS ONE* for specific examples.	The authors have declared that no competing interests exist

NO authors have competing interests

Enter: *The authors have declared that no competing interests exist.*

Authors with competing interests

Enter competing interest details beginning with this statement:

I have read the journal's policy and the authors of this manuscript have the following competing interests: [insert competing interests here]

* typeset

Ethics Statement

Enter an ethics statement for this submission. This statement is required if the study involved:

- Human participants
- Human specimens or tissue
- Vertebrate animals or cephalopods
- Vertebrate embryos or tissues
- Field research

Write "N/A" if the submission does not require an ethics statement.

General guidance is provided below. Consult the submission guidelines for detailed instructions. **Make sure that all information entered here is included in the Methods section of the manuscript.**

Poznań University Review Board
KB 276/20
Form of constent obtained (written)

Powered by Editorial Manager® and ProduXion Manager® from Aries Systems Corporation

**Format for specific study types**<br><br>**Human Subject Research (involving human participants and/or tissue)**<br>• Give the name of the institutional review board or ethics committee that approved the study<br>• Include the approval number and/or a statement indicating approval of this research<br>• Indicate the form of consent obtained (written/oral) or the reason that consent was not obtained (e.g. the data were analyzed anonymously)<br><br>**Animal Research (involving vertebrate animals, embryos or tissues)**<br>• Provide the name of the Institutional Animal Care and Use Committee (IACUC) or other relevant ethics board that reviewed the study protocol, and indicate whether they approved this research or granted a formal waiver of ethical approval<br>• Include an approval number if one was obtained<br>• If the study involved *non-human primates*, add *additional details* about animal welfare and steps taken to ameliorate suffering<br>• If anesthesia, euthanasia, or any kind of animal sacrifice is part of the study, include briefly which substances and/or methods were applied<br><br>**Field Research**<br><br>Include the following details if this study involves the collection of plant, animal, or other materials from a natural setting:<br>• Field permit number<br>• Name of the institution or relevant body that granted permission	
**Data Availability**<br><br>Authors are required to make all data underlying the findings described fully available, without restriction, and from the time of publication. PLOS allows rare exceptions to address legal and ethical concerns. See the PLOS Data Policy and FAQ for detailed information.	Yes - all data are fully available without restriction

A Data Availability Statement describing where the data can be found is required at submission. Your answers to this question constitute the Data Availability Statement and **will be published in the article**, if accepted.<br><br>**Important:** Stating 'data available on request from the author' is not sufficient. If your data are only available upon request, select 'No' for the first question and explain your exceptional situation in the text box.<br><br>Do the authors confirm that all data underlying the findings described in their manuscript are fully available without restriction?	
**Describe where the data may be found in full sentences. If you are copying our sample text, replace any instances of XXX with the appropriate details.**<br><br>• If the data are **held or will be held in a public repository**, include URLs, accession numbers or DOIs. If this information will only be available after acceptance, indicate this by ticking the box below. For example: *All XXX files are available from the XXX database (accession number(s) XXX, XXX.).*<br>• If the data are all contained **within the manuscript and/or Supporting Information files**, enter the following: *All relevant data are within the manuscript and its Supporting Information files.*<br>• If neither of these applies but you are able to provide **details of access elsewhere**, with or without limitations, please do so. For example:<br><br>*Data cannot be shared publicly because of [XXX]. Data are available from the XXX Institutional Data Access / Ethics Committee (contact via XXX) for researchers who meet the criteria for access to confidential data.*<br><br>*The data underlying the results presented in the study are available from (include the name of the third party*	All relevant data are within the manuscript and its Supporting Information files.

and contact information or URL).<br>• This text is appropriate if the data are owned by a third party and authors do not have permission to share the data.<br><br>* typeset	
Additional data availability information:	

Powered by Editorial Manager® and ProduXion Manager® from Aries Systems Corporation

Click here to access/download;Manuscript;Mediating role of coping Wilski PLOS.docx

Impact of multiple sclerosis and self-management: mediating role played by coping strategies

Maciej Wilski[1*], Waldemar Brola[2], Maciej Tomczak[3]

[1]Department of Adapted Physical Activity, Poznań University of Physical Education, Poznan, Poland

[2] Collegium Medicum, Jan Kochanowski University, Kielce Poland

[3]Department of Psychology, Poznań University of Physical Education, Poznań, Poland

*Corresponding author: Maciej Wilski

Email: mwilski@wp.pl (MW)

Abstract

Low level of self-management in MS patients is considered to be a predominant factor that leads to poor rehabilitation efficacy. Studies focusing on the relationship between self-management and psychological variables that can be modified could contribute to expanding the knowledge needed to propose interventional programs aiming at patient activation. This study aimed to analyze whether coping strategies play a mediating role in the association between the perceived impact of multiple sclerosis (MS) and level of self-management in MS patients. The cross-sectional study included 382 MS patients. The patients completed the Multiple Sclerosis Self-Management Scale—Revised, Multiple Sclerosis Impact Scale-29, and Coping Inventory for Stressful Situations. The study hypothesis was evaluated using mediation analysis. The STROBE checklist specifically prepared for cross-sectional research was applied in this study for reporting. Results indicate that the emotion- and problem-focused strategies of coping can be treated as mediating the association between the MS impact and level of self-management in patients with MS. A negative relationship was found between the perceived MS impact and problem-oriented coping, while a positive relationship was found between problem-oriented coping and self-management. Furthermore, a positive relationship was found between the MS impact and emotion-oriented coping, while a negative relationship was found between emotion-oriented coping and self-management. The indirect role of the avoidance-oriented coping was not significant. Our study confirms the role played by coping strategies in patients' self-management. In MS, self-management determined by perceived MS impact can be controlled by decreasing emotional-coping while increasing problem-coping strategies. Our study imparts new knowledge regarding the potential interventions for improving the level of self-management in MS patients. It indicates that recognition of patients' illness perceptions as well as maladaptive coping strategies can help health professionals identify those who might be having lower level of self-management.

Introduction

Multiple sclerosis (MS) is an unpredictable, difficult-to-control disease with treatment options focusing mainly on symptom relief and delaying disease progression. Patients with MS may not realize the benefits of costly, inconvenient, and painful treatment procedures because these are intended to prevent relatively uncommon but disruptive events of relapse and progression of disability over the years [1]. For this reason, a significant number of patients do not participate in treatment programs for at least a certain period [2], which causes delays in the assessment of treatment effectiveness. Therefore, increasing patient participation in therapeutic programs is highly necessary to plan the future course of action. This can be achieved by encouraging patients to ensure active self-management of their disease. To date, patient participation is one of the greatest priorities of research conducted on MS [3].

Self-management signifies successful management in a disabling condition. It requires special skills supporting treatment through medication adherence, gathering information about illness and novel treatment options, participation in therapeutic decisions, performing self-care, maintaining social relationships, and finding emotional equilibrium [4,5]. Patient involvement in the form of high self-management skills is not sufficient for achieving a successful outcome, but it increases the chances of achieving a positive outcome. Recent studies provide significant evidence about the health advantages brought by self-management in people with MS [6,7]. It is associated with a reduction of fatigue [8], improvement in health-related quality of life [9], adherence to medication [10], and increase in physical functioning [11,12]. In the systematic review of Rae-Grant et al. [13], treatment outcomes in MS were shown to improve with the efficacy of self-management.

As self-management is the main element contributing to improving the effectiveness of MS treatment, the search for factors supporting its growth is one of the major research directions. So far, studies focused on determining the correlates of self-management have

revealed self-efficacy, realistic timeline, and treatment control perception as salient correlates of successful self-management in MS patients [14]. Other studies have indicated that MS patients, who have low support and lack socioeconomic resources, are likely to be associated with poor self-management [15]. Interestingly, no studies have dealt with the simple relationship between the patient's perceived state of the disease and his/her level of self-management, which is the core of the Common-Sense Model of Self-Regulation [16] emphasizing the role of health threat perception in regulating health behaviors.

Most self-regulatory theories assume that behavior is a consequence of personal experience [17]. When affected by a disease, patients develop certain perceptions about their health and attempt understanding and managing the problem [18]. Deteriorating health influences the patients to take intensified actions aimed at improving their health condition. In a situation when the disease does not lead to visibly negative effects, the individual does not need to act. This basic relationship is true in the case of predictable and controllable diseases. However, the scenario is different in some diseases like MS, in which the course of illness varies in time as well as between patients, is difficult to control, and the effects of self-management may not be evident for a long time. In such cases, the relationship between the perceived state of the disease and the actions taken to improve it may be conditioned by other factors. On the basis of the Common-Sense Model of Self-Regulation, an individual's perception of illness will be associated with self-management activities through coping strategies that serve as mediating variables.

Most models distinguish three primary dimensions of coping: problem-oriented coping, which involves a direct attempt for changing a stressful circumstance; emotion-oriented coping, which aims at lowering emotional distress; and avoidance-oriented coping, which includes searching for other people (social diversion) or keeping occupied with another task (distraction) [19]. These categories are not mutually exclusive. Although evidence shows that coping

strategies are stable [20], specific factors, such as the impact of a chronic illness, may change the preferred coping strategy, and consequently, the behavior. In the case of MS, coping has been proven to mediate the interaction between illness beliefs, mental and physical well-being [21], executive function and psychosocial adjustment [22], fatigue and the quality of life [23], disability and psychosocial loss [24], or perception of disease control and mental health [25]. However, the mediation role of coping in determining self-management behaviors has not yet been investigated.

Therefore, our study aimed to examine the relationship between the perceived impact of MS and level of self-management and to what extent the coping strategies act as mediators in this association. Since research regarding this topic is limited, no definite mediational hypotheses were formulated in the study.

Materials and methods

Design

The cross-sectional study included 382 MS patients who were enrolled with cooperation from two rehabilitation centers in Poland. The study protocol was compliant with the guidelines of the Helsinki Declaration (as revised in Brazil 2013). The inclusion criteria of the study included the following: positive MS diagnosis (McDonald's revised criteria), no relapse during the last 30 days prior to the enrollment, absence of any concomitant disease, and absence of cognitive problems and/or psychiatric disorders as reported by a neurologist. The patients were recruited during the control visit at the rehabilitation clinic by a research team member, between December 2015 and January 2017. After providing verbal instruction and explaining the study purpose, patients were asked if they were interested to participate. Those who were willing to participate were requested to provide informed consent in written form after assuring them that their data would remain confidential. Patients completed all the questionnaires in a calm room allotted especially for the needs of this study. If any missing information was found, patients

were asked to complete it. Furthermore, a research team member verified the data accuracy. Out of 400 participants, 18 were excluded because they did not complete the questionnaires. Ethical approval for the study was provided by the Institutional Review Board, and the study also complied with the STROBE (Strengthening the Reporting of Observational Studies in Epidemiology) checklist.

Measures

Self-management was measured with Multiple Sclerosis Self-Management Scale—Revised (MSSM-R) developed by Bishop and Frain [26] to perform a multidimensional assessment of the MS patients' behaviors and knowledge about self-management. This scale consists of a total of 24 items. A 5-point scale ranging from 1 (*Disagree completely*) to 5 (*Agree completely*) was used to rate the patient responses. Higher scores signify a greater level of self-management. The reliability as well as validity of MSSM-R was proven in an earlier study [14,15,27]. In this study, the MSSM-R scale indicated high internal consistency (Cronbach's α = 0.89).

The perceived level of MS impact was estimated using Multiple Sclerosis Impact Scale-29 (MSIS-29). This prominent self-report measure is used for assessing a patient's perception of the impact posed by MS on his/her life in the last 2 weeks [28]. The physical subscale used for assessment included 20 questions evaluating the functional ability of patients, with scores ranging from 20 to 100. Higher scores signify worse perception of patients about their physical condition. Studies have shown that the MSIS-29 subscale is a sensitive, accurate, and valid tool for assessing MS populations [29,30]. In this study, the MSIS-29 scale showed high internal consistency (Cronbach's α = 0.98).

Coping strategies were assessed with Coping Inventory for Stressful Situations (CISS) developed by Endler and Parker [31]. This is a 48-item measure that evaluates the following styles of coping: problem-oriented, emotion-oriented, and avoidance-oriented. The score for

each scale ranges between 16 and 80. The responses are indicated by a Likert-type 5-point scale, ranging from 1 (*Not at all*) to 5 (*Very much*). Higher scores indicate a greater level of coping. The CISS scale is used for assessing different illness groups as its validity and reliability are high [31]. In this study, the CISS scale showed high internal consistency (the α value for problem-oriented, emotion-oriented, and avoidance-oriented style of coping was 0.79, 0.88, and 0.69, respectively).

We used a validated questionnaire in this study to collect demographic and clinical information of the patients. All patients were requested to provide data about age, sex, education, occupation, marital status, disease duration, and diagnosed MS type. In addition, the neurologist assessed the severity level of MS in patients using expanded disability status scale (EDSS), which ranges between 0 (*Normal examination*) and 10 (*Death from MS*) [32], and reported whether the patient had any cognitive and/or psychiatric problems.

Statistical analysis

To determine if the coping strategy used to deal with stress (problem-, emotion-, and avoidance-oriented style of coping) mediates the association between the MS impact (independent variable) and self-management (dependent variable), we performed mediation analysis. Due to the potential importance of disability index (EDSS) and the time since diagnosis of patients' functioning, these variables were included in the presented model as covariates (especially for self-management level, the correlation with EDSS and time from diagnosis was: $r=-0.28$, $p<0.001$, bootstrap percentile 95%CI [-.374; -.184] and $r=-0.27$, $p<0.001$, 95%CI [-.364; -.187] respectively). To determine the significance of indirect effects, we calculated a 95% confidence interval (CI) with percentile bootstrapping (for estimating the standard error and CI, we used 50000 samples). The statistical analysis was conducted using the PROCESS macro developed by Hayes [33].

Results

The patients had a mean age of 46 years (126 men and 256 women). With regard to the marital status, more than 32% of the patients were single, divorced, or widowed and 67% were married. With regard to the employment status, 38% were employed and 56% were either retired or were receiving a disability pension. The mean disease duration was 11 years, and the majority of MS patients showed a disease characterized by relapsing–remitting course (41%). The disability level (measured with EDSS) was mild and moderate in more than 90%, and only less than 10% of the patients had a higher level of disability. Table 1 summarizes the sociodemographic and clinical characteristics of the patients.

Table 1. Sociodemographic and illness characteristic of the MS study sample.

Sociodemographic and illness characteristic	Patients with MS (N = 382)
Age (years) at the time of participation in this study (mean ± SD; min–max)	46.4 ± 11.9 (18–82)
Gender n (%)	
Female	256 (67.0)
Male	126 (33.0)
Marital status n (%)	
Single	71 (18.6)
Married	257 (67.3)
Separated/Divorced/Widowed	55 (14.1)
Employment n (%)	
Employed	146 (38.2)
Unemployed	21 (5.5)
Disability pension/retiring	215 (56.3)
Time (years) since the diagnosis of MS (mean ± SD; min–max)	11.6 ± 9.1 (1–48)
Diagnosed type of MS n (%)	
Relapsing–remitting (RRMS)	158 (41.4)
Primary progressive (PPMS)	88 (23.0)
Secondary progressive (SPMS)	70 (18.3)
Progressive–relapsing (PRMS)	33 (8.6)
Don't know (DKMS)	33 (8.6)
Disability subgroups "EDSS" n (%)	
Mild (EDSS ≤ 3.5)	166 (43.5)
Moderate (3.5 < EDSS ≤ 6.5)	181 (47.4)
High (EDSS < 6.5)	35 (9.2)

Abbreviations: EDSS, expanded disability status scale; SD, standard deviation; MS, multiple sclerosis; CI, confidence interval.

Mediation analysis

Statistically significant indirect effects were obtained for problem-oriented coping (-.01384, [95% CI: -.03075; -.00063]; standardized effect: -.02831, [95% CI: -.06460; -.00125]) and emotion-oriented coping (-.03528, [95% CI: -.06093; -.01208]; standardized effect: -.07219, [95% CI: -.12558; -.02479]) (Table 2). A negative association was found between the MS impact and problem-oriented coping ($B=-0.07$, $p<0.001$), while a positive association was found between problem-oriented coping and patient's self-management ($B=0.20$, marginal significant effect: $p=0.0539$). In addition, a positive association was found between the MS impact and emotion-oriented coping ($B=0.16$, $p<0.001$), and a negative association was found between emotion-oriented coping and patient's self-management ($B=-0.22$, $p<0.01$) (Fig 1).

Fig 1. Coping strategies [problem-oriented (P), emotion-oriented (E), and avoidance-oriented (A)] as mediators between the impact of multiple sclerosis (MSI) and patients' self-management (SM) with expanded disability status scale (EDSS) and time from diagnosis (TD) as covariates.

Indirect effect for avoidance-oriented coping did not achieve statistical significance (-.01097, [95% CI: -.02788; .00007]; standardized effect: -.02245 [95% CI: -.05579; .00015]) (Table 2). Both total ($B=-0.02$, $p>0.05$) and direct effects ($B=0.04$, $p>0.05$) between the MS impact and patient's self-management did not reach statistical significance.

Table 2. Indirect effects for mediational relations between the impact of the disease, coping strategies, and self-management in patients with MS.

Potential mediator	Indirect effect [95% CI]	Standardized indirect effect [95% CI]
Problem-oriented coping	-.01384 [-.03075 -.00063]	-.02831 [-.06460 -.00125]
Emotion-oriented coping	-.03528 [-.06093 -.01208]	-.07219 [-.12558 -.02479]
Avoidance-oriented coping	-.01097 [-.02788 .00007]	-.02245 [-.05579 .00015]
**Total**	-.06009 [-.09120 -.03188]	-.12295 [-.18850 -.06511]

Abbreviations: MS, multiple sclerosis; CI, confidence interval.

Discussion

As far as we know, this study was the first to analyze whether coping strategies act as a mediator in the association between the perceived MS impact and the level of self-management in MS patients. Our results provide the following conclusions. First, no significant relationship was found between the perceived MS impact and the level of self-management under control of time from diagnosis and disability level; however, a relationship was realized through mediation. Second, patients with a worse perception of MS used more emotion-coping strategies, and this in turn led to a lower level of self-management under control of time from diagnosis and disability level. Third, a negative association was found between the MS impact and problem-oriented coping, while a positive association was observed between problem-oriented coping and self-management under control of time from diagnosis and disability level. Finally, the mediating effect was not observed for avoidance-oriented coping.

According to our studies, in the case of MS, perception of the disease does not directly translate into a patient's activity in the form of self-management; however, a part of this relationship is mediated by coping practices. This means that the patient's perception of the MS severity guides him/her to take up a coping strategy, which in turn influences the self-management activity. This agrees with the speculative assumptions of the Common-Sense Model of Self-Regulation.

Specifically, our results indicate that patients with a worse perception about the MS impact tend to apply emotion-oriented coping strategies, which in turn leads to a low level of self-management. This seems reasonable as earlier studies have demonstrated that health deterioration of an MS patient is associated with depression and due to applying less adaptive emotion-centered coping [34]. Other studies have reported that higher disability in MS patients is linked with high scores on depression scales and these patients display less flexible, emotion-focused strategies of coping [35]. This clearly shows that worse perception about the impact of MS negatively influences the psychological health of patients. As a result, the patients apply emotional strategies, which consequently affect their adjustment [36], quality of life [37], and level of self-management (as revealed in this study).

Problem-oriented coping also mediated the association between the perceived MS impact and the level of self-management. These coping strategies are generally considered to be more adaptive and by definition are related to active participation in the treatment process. Our study showed that MS patients who have a more favorable perception of the disease use more adaptive problem-coping strategies, which leads to more effective self-management. However, empirical evidence from earlier studies has revealed mixed findings. For example, in a previous study, problem-oriented coping strategies were unrelated to adjustment indicators [38], whereas in another study, the results showed that these strategies were related to better psychological health [21,25]. Some researchers indicate that in the case of conditions that could not be controlled by behavioral changes or treatment, problem-oriented coping is of no value [39-41]. However, our study corroborates the hypothesis that a greater focus on problem-oriented coping strategies translates into a higher level of self-management in patients with MS.

Despite the significant relationships between the avoidance-oriented coping, perceived impact of MS, and level of self-management, no significant indirect effect was observed, which indicates that avoidance is not a mediator in the analyzed relationship. The efficacy of

avoidance-oriented coping in scientific research remains unclear due to divergent results. Most of the findings report that avoidance-oriented coping is maladaptive, which translates to worse adjustment in MS patients [42,43]; however, some studies indicate that avoidance can be applied as a kind of adaptive approach for a certain period to avert negative perception about future MS progression and prevent depression [23,44,45]. A possible explanation of this contradiction might be that there are differences in the definition of avoidance-oriented coping. For example, in the case of the frequently used Brief COPE questionnaire, avoidance means denial, behavioral disengagement, and substance use. In our study, we used the CISS questionnaire, which defines avoidance as activities or cognitive modifications adapted to avoid a stressful circumstance, which is achieved through diverting to other situations or engaging oneself in or other tasks (task-oriented) or by social diversion (person-oriented) for reducing stress. Such defined avoidance does not have to be nonadaptive. Invariably, our results do not clarify the role of avoidance in shaping self-management, which certainly needs further in-depth research.

In addition, our results provide evidence that the level of self-management decreases with higher disability and a longer duration of the illness. This is contradictory to our earlier reports [14,15], but the difference may stem from the variations in the examined groups. Our study sample included a significantly higher percentage of patients with mild EDSS (≤3.5; 43%) than that of previous studies (11% and 8%, respectively). There is a need for research on a more representative group in terms of disability, which may explain the discrepancy in results.

Despite the need for longitudinal studies for proving causality, the results of our study are of practical value to MS patients as well as health professionals dealing with them. First, patients with MS and health professionals should be educated on the effect of negative illness perception and emotion coping strategies on self-management. Second, actions directed at ways of coping should be indicated, because they mediate the level of self-management. Special

attention should be paid to lowering the emotional techniques of coping and motivating patients to engage in problem-solving coping. Although these relationships are known in relation to other forms of activity of people with various diseases, the present study for the first time indicates their importance in self-management of patients with MS, which is an uncertain, unpredictable, and difficult-to-manage disease. Third, specific strategies are triggered as a consequence of disease perception. Therefore, appraisal of MS impact needs to be regularly examined in individuals, particularly in those showing poor involvement in the treatment process. Exaggerating the risk and complications of MS might lead to choosing less adaptive, more emotion-oriented coping strategies and, as a consequence, reduce self-management activities. Challenging the negative perception of MS impact and developing a positive perception could promote more adaptive forms of coping. This can be achieved through offering objective data on the characteristics of the illness and its progression, as well as promoting cognitive changes by eliminating negative beliefs and strengthening positive thoughts. Due to frequent contact with patients, nursing staff play a special role in this respect. These interventions could help in the proposal of patient-focused self-management approaches and be advantageous to medical programs treating MS patients.

The present study has some limitations which should be taken into account when interpreting the results. First, this is a cross-sectional study, which does not allow any conclusion to be made regarding the directionality of the associations. Therefore, longitudinal studies should be carried out to provide additional insights on the mediation patterns reported here. Second, the data examined in the study were self-reported by patients, which indicates the possibility of response bias as well as shared method bias. Third, the majority of patients (91%) showed mild and moderate disability (EDSS ≤6.5), while only 9% showed severe disability, which limits the possibilities about the generalization of the results. In addition, we excluded patients with cognitive problems through subjective neurological examination unsupported by

an objective research instrument. This can undermine the applied exclusion criterion. Finally, we cannot exclude other variables that have a potential relationship with the level of self-management in MS patients. Further research should be focused on identifying those factors whose modifications will allow developing self-management skills.

Conclusion

The findings of our study highlight coping strategies as a modifiable factor of the association between the perceptions of MS impact and the level of self-management. Specifically, with a worse perception of the disease, patients tend to use more emotion-centered coping, which negatively influences their self-management. On the other hand, with a better perception of the disease, patients use more problem-oriented strategies, which is associated with a higher level of self-management. Thus, lowering the emotional response and increasing problem-solving attitude promote activities related to the involvement of an MS patient in the process of treatment and rehabilitation.

Acknowledgements

We would like to gratefully acknowledge all the individuals with multiple sclerosis who completed a questionnaire and Dr. Mariusz Kowalewski, the manager of Multiple Sclerosis Rehabilitation Centre in Borne Sulinowo for his help in enrollment of the study participants.

References

1. Steinberg S, Faris R, Chang C, Chan A, Tankersley M. Impact of Adherence to Interferons in the Treatment of Multiple Sclerosis. Clin Drug Investig. 2010;30(2):89-100. DOI: 10.2165/11533330-000000000-00000
2. Henze T, Rieckmann P, Toyka K. Symptomatic Treatment of Multiple Sclerosis. Eur Neurol. 2006;56(2):78-105.

3. Rieckmann P, Boyko A, Centonze D, Elovaara I, Giovannoni G, Havrdová E et al. Achieving patient engagement in multiple sclerosis: A perspective from the multiple sclerosis in the 21st Century Steering Group. Mult Scler Relat Disord. 2015;4(3):202-218. DOI: 10.1016/j.msard.2015.02.005
4. Kralik D, Koch T, Price K, Howard N. Chronic illness self-management: taking action to create order. J Clin Nurs. 2004;13(2):259-267. DOI: 10.1046/j.1365-2702.2003.00826.x
5. Bishop M, Frain M, Tschopp M. Self-Management, Perceived Control, and Subjective Quality of Life in Multiple Sclerosis. Rehabil Couns Bull. 2008;52(1):45-56. DOI: 10.1177/0034355208320000
6. Plow M, Finlayson M, Rezac M. A Scoping Review of Self-Management Interventions for Adults With Multiple Sclerosis. PM&R. 2011;3(3):251-262. DOI: 10.1016/j.pmrj.2010.11.011
7. Kidd T, Carey N, Mold F, Westwood S, Miklaucich M, Konstantara E et al. A systematic review of the effectiveness of self-management interventions in people with multiple sclerosis at improving depression, anxiety and quality of life. PLoS ONE. 2017;12(10):e0185931. DOI: 10.1371/journal.pone.0185931
8. Navipour H, Madani H, Mohebbi M, Navipour R, Roozbayani P, Paydar A. Improved fatigue in individuals with multiple sclerosis after participating in a short-term self-care programme. NeuroRehabilitation. 2006;21(1):37-41. DOI: 10.3233/NRE-2006-21106
9. Jongen P, Wesnes K, van Geel B, Pop P, Schrijver H, Visser L et al. Does Self-Efficacy Affect Cognitive Performance in Persons with Clinically Isolated Syndrome and Early Relapsing Remitting Multiple Sclerosis?. Mult Scler Int. 2015;2015:1-8. DOI: 10.1155/2015/960282

10. Berger B, Liang H, Hudmon K. Evaluation of Software-Based Telephone Counseling to Enhance Medication Persistency Among Patients with Multiple Sclerosis. J Am Pharm Assoc. 2005;45(4):466-472. DOI: 10.1331/1544345054475469
11. McAuley E, Motl R, Morris K, Hu L, Doerksen S, Elavsky S et al. Enhancing physical activity adherence and well-being in multiple sclerosis: a randomised controlled trial. Mult Scler J. 2007;13(5):652-659. DOI: 10.1177/1352458506072188
12. Bombardier C, Cunniffe M, Wadhwani R, Gibbons L, Blake K, Kraft G. The Efficacy of Telephone Counseling for Health Promotion in People With Multiple Sclerosis: A Randomized Controlled Trial. Arch Phys Med Rehabil. 2008;89(10):1849-1856. DOI: 10.1016/j.apmr.2008.03.021
13. Rae-Grant A, Turner A, Sloan A, Miller D, Hunziker J, Haselkorn J. Self-management in neurological disorders: Systematic review of the literature and potential interventions in multiple sclerosis care. J Rehabil Res Dev. 2011;48(9):1087. DOI: 10.1682/jrrd.2010.08.0159
14. Wilski M, Tasiemski T. Illness perception, treatment beliefs, self-esteem, and self-efficacy as correlates of self-management in multiple sclerosis. Acta Neurol Scand. 2016;133(5):338-345. DOI: 10.1111/ane.12465
15. Wilski M, Tasiemski T, Kocur P. Demographic, socioeconomic and clinical correlates of self-management in multiple sclerosis. Disabil Rehabil. 2015;37(21):1970-1975. DOI: 10.3109/09638288.2014.993435
16. Leventhal H, Brissette I, Leventhal EA. The common-sense model of self-regulation of health and illness. In: Cameron LD, Leventhal H, editors. The self-regulation of health and illness behavior. Routledge; 2003. pp. 43-65.
17. Cameron LD, Leventhal H, editors. The self-regulation of health and illness behaviour. Psychology press; 2003.

18. Leventhal H, Meyer D, Nerenz D. The common sense representations of illness danger. In: Rachman S, editor. Medical psychology. New York: Pergamon; 1980. Vol. 2, pp. 7–30.
19. Parker JDA, Endler NS. Coping and defense: A historical overview. In: Zeidner M, Endler NS, editors. Handbook of coping: Theory, research, applications. New York: Wiley; 1996. pp. 3–23.
20. Gil K, Wilson J, Edens J. The Stability of Pain Coping Strategies in Young Children, Adolescents, and Adults with Sickle Cell Disease Over an 18-Month Period. Clin J Pain. 1997;13(2):110-115. DOI: 10.1097/00002508-199706000-00005
21. Bassi M, Cilia S, Falautano M, Grobberio M, Niccolai C, Pattini M et al. Illness perceptions and psychological adjustment among persons with multiple sclerosis: the mediating role of coping strategies and social support. Disabil Rehabil. 2019;:1-13. DOI: 10.1080/09638288.2019.1610511
22. Grech L, Kiropoulos L, Kirby K, Butler E, Paine M, Hester R. Target Coping Strategies for Interventions Aimed at Maximizing Psychosocial Adjustment in People with Multiple Sclerosis. Int J MS Care. 2018;20(3):109-119. DOI: 10.7224/1537-2073.2016-008
23. Mikula P, Nagyova I, Krokavcova M, Vitkova M, Rosenberger J, Szilasiova J et al. The mediating effect of coping on the association between fatigue and quality of life in patients with multiple sclerosis. Psychol Health Med. 2015;20(6):653-661. DOI: 10.1080/13548506.2015.1032310
24. Gedik Z, Sorias O, Idiman E. Do coping styles mediate the relationship between disability status and psychosocial loss in people with relapsing remitting multiple sclerosis?. J Health Psychol. 2015;22(6):707-721. DOI: 10.1177/1359105315611958
25. Wilski M, Brola W, Tomczak M. Health locus of control and mental health in patients with multiple sclerosis: Mediating effect of coping strategies. Res Nurs Health. 2019;42(4):296-305. DOI: 10.1002/nur.21955

26. Bishop M, Frain M. The Multiple Sclerosis Self-Management Scale: Revision and psychometric analysis. Rehabil Psychol. 2011;56(2):150-159. DOI: 10.1037/a0023679
27. Ghahari S, Khoshbin L, Forwell S. The Multiple Sclerosis Self-Management Scale. Int J MS Care. 2014;16(2):61-67. DOI: 10.7224/1537-2073.2013-019
28. Hobart J. The Multiple Sclerosis Impact Scale (MSIS-29): A new patient-based outcome measure. Brain. 2001;124(5):962-973. DOI: 10.1093/brain/124.5.962
29. Hoogervorst E, Zwemmer J, Jelles B, Polman C, Uitdehaag B. Multiple Sclerosis Impact Scale (MSIS-29): relation to established measures of impairment and disability. Mult Scler J. 2004;10(5):569-574. DOI: 10.1191/1352458504ms1078oa
30. Riazi A, Hobart J, Lamping D, Fitzpatrick R, Thompson A. Evidence-based measurement in multiple sclerosis: the psychometric properties of the physical and psychological dimensions of three quality of life rating scales. Mult Scler J. 2003;9(4):411-419. DOI: 10.1191/1352458503ms929oa
31. Endler NS, Parker JDA. The Coping Inventory for Stressful Situations (CISS): Manual. 2nd ed. Toronto: Multi-Health Systems; 1999.
32. Kurtzke J. Rating neurologic impairment in multiple sclerosis: An expanded disability status scale (EDSS). Neurology. 1983;33(11):1444-1452. DOI: 10.1212/WNL.33.11.1444
33. Hayes AF. Introduction to mediation, moderation, and conditional process analysis: A regression-based approach. New York: The Guilford Press; 2013.
34. Kroencke D, Denney D. Stress and coping in multiple sclerosis: exacerbation, remission and chronic subgroups. Mult Scler J. 1999;5(2):89-93. DOI: 10.1177/135245859900500204
35. Mohr D, Goodkin D, Gatto N, Van Der Wende J. Depression, coping and level of neurological impairment in multiple sclerosis. Mult Scler J. 1997;3(4):254-258. DOI: 10.1177/135245859700300408

36. Pakenham K. Adjustment to multiple sclerosis: Application of a stress and coping model. Health Psychol. 1999;18(4):383-392. DOI: 10.1037/0278-6133.18.4.383
37. McCabe M. A Longitudinal Study of Coping Strategies and Quality of Life Among People with Multiple Sclerosis. J Clin Psychol Med Settings. 2006;13(4):367-377. DOI: 10.1007/s10880-006-9042-7
38. Pakenham K. Investigation of the coping antecedents to positive outcomes and distress in multiple sclerosis (MS). Psychol Health. 2006;21(5):633-649. DOI: 10.1080/14768320500422618
39. Affleck G, Tennen H, Pfeiffer C, Fifield J. Appraisals of control and predictability in adapting to a chronic disease. J Pers Soc Psychol. 1987;53(2):273-279. DOI: 10.1037/0022-3514.53.2.273
40. Hagger M, Orbell S. A Meta-Analytic Review of the Common-Sense Model of Illness Representations. Psychol Health. 2003;18(2):141-184. DOI: 10.1080/0887044031000081321
41. Willemse H, van der Doef M, van Middendorp H. Applying the Common Sense Model to predicting quality of life in alopecia areata: The role of illness perceptions and coping strategies. J Health Psychol. 2018;24(11):1461-1472. DOI: 10.1177/1359105317752826
42. Dennison L, Moss-Morris R, Chalder T. A review of psychological correlates of adjustment in patients with multiple sclerosis. Clin Psychol Rev. 2009;29(2):141-153. DOI: 10.1016/j.cpr.2008.12.001
43. Bassi M, Grobberio M, Negri L, Cilia S, Minacapelli E, Niccolai C et al. The Contribution of Illness Beliefs, Coping Strategies, and Social Support to Perceived Physical Health and Fatigue in Multiple Sclerosis. J Clin Psychol Med Settings. 2019;.1-12. DOI: 10.1007/s10880-019-09692-6

44. Calandri E, Graziano F, Borghi M, Bonino S. Coping strategies and adjustment to multiple sclerosis among recently diagnosed patients: the mediating role of sense of coherence. Clin Rehabil. 2017;31(10):1386-1395. DOI: 10.1177/0269215517695374

45. Dennison L, Yardley L, Devereux A, Moss-Morris R. Experiences of adjusting to early stage Multiple Sclerosis. J Health Psychol. 2010;16(3):478-488.

Click here to access/download;Figure;Figure-1.tiff

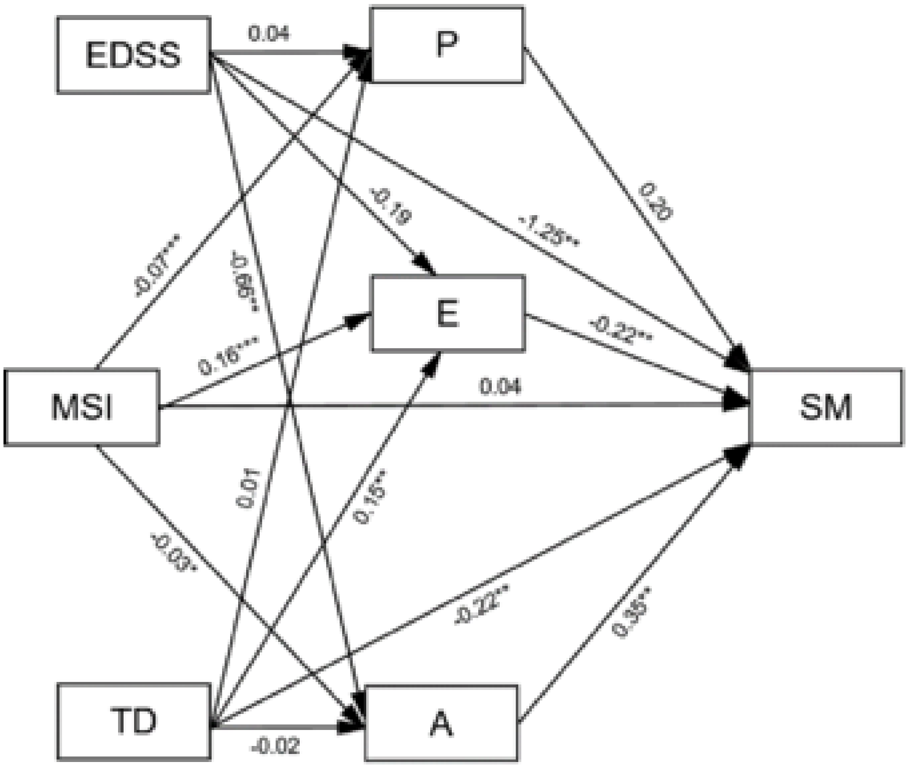

Data

Click here to access/download
Supporting Information
Data PLOS Wilski.xlsx
