# Supplementary material for: The perceived impact of multiple sclerosis and self-management: The mediating role of coping strategies
Source: PLoS One. 2021 Mar 11;16(3):e0248135. doi: 10.1371/journal.pone.0248135 (PMC7951896; doi:10.1371/journal.pone.0248135)
Supplement: Attachment — Submitted filename: Response to Reviews 3.docx [file pone.0248135.s004.docx]

**Response to Reviewers**

**“The perceived impact of MS and self-management: The mediating role of coping strategies”**

Based on the valuable guidelines we have made some changes in the text. We hope that the end result is satisfactory for the reviewers and editors.

***Editor*:**

***P5 line 100 shown not proven***

***P6 line 129 "the Polish”***

***P11 Line 236. You provide a good answer to Reviewer 1’s question about conducting mediation analysis. I think this issue may be in the minds of other readers too. So please very briefly note that mediation is an appropriate analysis approach***

***P13 Line 296 testing, not proving***

***While the language on causality may have been revised, the interpretation in this paragraph still assumes causality based on theoretical grounds. For example, it is suggested that "people with MS and health professionals should be educated on the effect of negative illness perception and emotion coping strategies on self-management.” This assumes a direction of effect, when it could be, for example, that coping strategy drives illness perception rather than vice versa. It is fine to base the interpretation on theoretical grounds, but it needs to be clear that is what you are doing and that longitudinal models will be helpful in testing whether that interpretation is indeed correct. So please consider revising the discussion to address this point further***

We are very grateful for all the suggestions. We have improved the text in accordance with all of your instructions.
